# Gynecologists’ perspectives on surgical treatment for apical prolapse: a qualitative study

**DOI:** 10.1007/s00192-023-05587-1

**Published:** 2023-07-01

**Authors:** Anique M. J. van Oudheusden, Mirjam Weemhoff, Leah F. Menge, Brigitte A. B. Essers

**Affiliations:** 1 Department of Gynecology and Obstetrics, VieCuri Medical Centre, P.O. Box 1926, 5900 BX Venlo, The Netherlands; 2 Department of Gynecology and Obstetrics, GROW, School for Oncology & Reproduction, Maastricht University, P.O. Box 616, 6200 MD Maastricht, The Netherlands; 3 Department of Gynecology and Obstetrics, Zuyderland Medical Centre, P.O. Box 5500, 6130 MB Sittard-Geleen, The Netherlands; 4 Department of Gynecology and Obstetrics, Reinier de Graaf Hospital, P.O. Box 5011, 2600 GA Delft, The Netherlands; 5 Department of Clinical Epidemiology and Medical Technology Assessment (KEMTA), Maastricht University Medical Centre, P.O. Box 616, 6200 MD Maastricht, The Netherlands

**Keywords:** Vaginal vault prolapse, Uterine descent, Sacrocolpopexy, Sacrohysteropexy, Sacrospinous fixation

## Abstract

**Introduction and hypothesis:**

Vaginal sacrospinous fixation (VSF) without mesh and sacrocolpopexy (SCP) with mesh are the most frequently performed surgical procedures for apical prolapse in the Netherlands. There is no long-term evidence suggesting the optimal technique, however. The aim was to identify which factors play a role in the choice between these surgical treatment options.

**Methods:**

A qualitative study using semi-structured interviews amongst Dutch gynecologists was carried out. An inductive content analysis was performed with Atlas.ti.

**Results:**

Ten interviews were analyzed. All gynecologists performed vaginal surgeries for apical prolapse, six gynecologists perform SCP themselves. Six gynecologists would perform VSF for a primary vaginal vault prolapse (VVP); three gynecologists preferred a SCP. All participants prefer a SCP for recurrent VVP. All participants have stated that multiple comorbidities could be a reason for choosing VSF, as this procedure is considered less invasive. Most participants choose a VSF in the case of older age (6 out of 10) or higher body mass index (7 out of 10). All treat primary uterine prolapse with vaginal, uterine-preserving surgery.

**Conclusions:**

Recurrent apical prolapse is the most important factor in advising patients which treatment they should undergo for VVP or uterine descent. Also, the patient’s health status and the patient’s own preference are important factors. Gynecologists who do not perform the SCP in their own clinic are more likely to perform a VSF and find more reasons not to advise a SCP. All participants prefer a vaginal surgery for a primary uterine prolapse.

**Supplementary information:**

The online version contains supplementary material available at 10.1007/s00192-023-05587-1

## Introduction

Pelvic organ prolapse (POP) is a frequently occurring health issue and is expected to increase as age and obesity rates are rising [1]. The lifetime risk of women undergoing surgery for POP has reported to be 20% by the age of 80 years [2]. Traditionally, uterine prolapse has been treated with vaginal hysterectomy (VH). Interest in uterine-preserving treatments is gaining, as more literature on this matter has been published [3–7]. Women prefer uterus-preserving prolapse surgery in the absence of substantial benefit of VH [8]. Besides POP, there are other reasons for performing a hysterectomy, including heavy menstrual bleeding or cervix dysplasia. The prevalence of vaginal vault prolapse (VVP) in women who underwent a VH for pelvic organ prolapse has been reported in 23% of the cases. In women who had a hysterectomy for another reason, laparoscopically or vaginally, the prevalence of VVP was 4.4% and 5.8% respectively [9].

Vaginal sacrospinous fixation (VSF) without mesh and sacrocolpopexy (SCP) with mesh are the most frequently performed surgical procedures for apical prolapse in the Netherlands [10]. Several randomized trials have been conducted to compare laparoscopic and vaginal treatments for apical prolapse. The LAVA trial (*n* = 126) showed the non-inferiority of the laparoscopic sacrohysteropexy (LSH) compared with the vaginal sacrospinous hysteropexy (SSHP), in the treatment of uterine descent, for bothersome bulge symptoms, after 12 months of follow-up [4]. The results of the VUE study (vault trial, *n* = 208) showed no differences between the laparoscopic sacrocolpopexy (LSC) and the VSF, as treatment for VVP, in terms of efficacy, quality of life, or adverse events at 1-year follow-up [11]. The SALTO-2 trial (*n* = 64) also reported no differences between LSC and VSF on anatomical and functional outcome, in the treatment of VVP, at 1-year follow-up. This trial, however, intended to include 106 women and was stopped prematurely. The main reason for not including the targeted sample size was a patients’ preference for one of the two surgeries [12, 13].

Laparoscopic sacrocolpopexy and VSF are very different procedures; thus, it is conceivable that gynecologists also have a certain preference. A previously conducted clinical practice survey showed no standardized method for the treatment of VVP. The VSF appeared to be the first-choice treatment of VVP among Dutch gynecologists [10]. However, it is unclear what factors contribute to their preference. There are no specific patient characteristics known to favor one technique over the other. The aim of this qualitative study was to identify the factors that influence Dutch gynecologists in making their choice and counseling patients.

## Materials and methods

### Study design

A qualitative study consisting of semi-structured interviews was conducted amongst Dutch gynecologists. All interviews were performed by one researcher (AVO); no other people were present during the interviews. The researcher knew some of the participants, but there was no work relationship or dependency of any sort. Participants were contacted by e-mail and the interview took place by a 20- to 30-min videoconference call. Respondents were asked beforehand, with a short digital questionnaire, which surgeries they performed; how many of those surgeries they perform per year; and how many procedures they had carried out in total. In order to represent various perspectives on this matter gynecologists were selected in different types of hospitals, i.e., university hospitals, non-university teaching hospitals, and non-university nonteaching hospitals.

The Medical Ethical Research Committee of the Zuyderland Medical Centre (METC Z) exempted the need for ethical review (file number METCZ20220042), because it was not subject to the Medical Research Involving Human Subjects Act [14]. This study was developed and described in accordance with the consolidated criteria for reporting qualitative research [15].

### Data collection

A list of topics was drawn up and tested in a pilot interview (AVO, MW, and LM), prior to the start of the study. Minor alterations were made and then the study started. At the start of each interview, the participant was asked what their first-choice surgical treatment for primary and recurrent VVP would be. Open-ended questions were used to explore the experiences of the participant and investigate which factors were important in deciding on the optimal surgical treatment. Last, participants were asked to give their top five factors that were of importance in their decision.

### Data processing and analysis

The interviews were recorded and thereafter fully transcribed, with the use of Amberscript.com. The transcripts were coded and analyzed by two researchers (AVO and LM), using the qualitative analyzing tool Atlas.ti 9.0.23 for Windows. Disagreements during coding were few and were discussed until consensus was reached between the two researchers. Data are presented as numbers and are discussed qualitatively. The top five factors were reversely scored, 5 points for the most important factor, 4 points for the second most important factor, and so on.

## Results

Saturation of data was reached after eight interviews, i.e., no new factors were mentioned during the interviews. Two additional interviews were held to confirm saturation. In total, ten gynecologists participated in the study. No one declined participation or dropped out prematurely. Characteristics of respondents are shown in Table 1. Four of the participants were subspecialized urogynecologists. All gynecologists perform post-hysterectomy sacrospinous fixation (VSF), vaginal sacrospinous hysteropexy (SSHP), and VH. SCP and sacrohysteropexy (SHP) are performed by six participants themselves, laparoscopically or robot assisted. Three gynecologists have to refer to another hospital for SCP and SHP.

**Table 1:** Characteristics of respondents

	Respondents (*n* = 10)
Sex	
Female	7/10
Male	3/10
Age (years), median (IQR)	50.5 (45.5–52.5)
Hospital type	
Academic teaching hospital	2/10
Non-academic teaching hospital	6/10
Non-academic nonteaching hospital	2/10
Experience as a gynecologist (years), median (IQR)	13.5 (8.5–16.3)
Specialty	
Urogynecologist	4/10
Gynecologist with urogynecological focus	6/10
Procedure performed	
VSF/SSHP/VH	10/10
Modified Manchester	9/10
Sacrocolpopexy/sacrohysteropexy performed by participant^a^	6/10
Sacrocolpopexy/sacrohysteropexy performed by colleague^a^	1/10
No sacrocolpopexy/sacrohysteropexy performed in hospital^a^	3/10
Combining laparoscopic/vaginal surgery (perineoplasty)^b^	3/10
Procedures performed in total	
VSF	
1–100	3/10
> 100	7/10
SSHP	
1–100	2/10
> 100	8/10
Modified Manchester	
1–100	4/10
> 100	6/10
Vaginal hysterectomy	
1–100	2/10
> 100	8/10
LSC/LSH	
0	4/10
1–100	5/10
> 100	1/10
RSC/RSH	
0	9/10
1–100	–
> 100	1/10

No./no. of total respondents, unless stated otherwise

*VSF* vaginal sacrospinous fixation; *SSHP* vaginal sacrospinous hysteropexy, *VH* vaginal hysterectomy, *LSC* laparoscopic sacrocolpopexy, *RSC* robotic sacrocolpopexy, *LSH* laparoscopic sacrohysteropexy, *RSH* robotic sacrohysteropexy

^a^Sacrocolpopexy and sacrohysteropexy, performed per laparoscopy, robot, or abdominally

^b^Combining sacrocolpopexy or sacrohysteropexy with vaginal surgery for pelvic organ prolapse

All topics were categorized in the following themes: type of prolapse, patient-related factors, surgery-related factors, and physician-related factors. The code tree with themes, subthemes, and topics is presented in Supplementary Fig. 1.

### Preferred treatment for vaginal vault prolapse

Table 2 shows the first-choice treatment for the different types of prolapse for all gynecologists and subdivided into groups depending on whether they have to refer to another hospital for this procedure. Six gynecologists would perform a VSF for a primary VVP; 3 gynecologists preferred a SCP; and 1 gynecologist has no specific preference. When the previous hysterectomy was carried out as treatment for uterine prolapse, 2 gynecologists would still advise a VSF, whereas 5 other participants would prefer a SCP. All participants would prefer a SCP after a previous treatment for VVP. Whether the VVP is a recurrent apical prolapse or a recurrent VVP is the most important factor for most participants (Table 3).

**Table 2:** First-choice surgical treatment for the type of prolapse

Surgical treatment of first choice for VVP	VSF	LSC/RSC	No preference
VVP after hysterectomy for a reason other than prolapse	6/10	3/10	1/10
Performing SC/SC in the same hospital	3/10	3/10	1/10
Referring to another hospital for SC	3/10	*–*	*–*
VVP after hysterectomy for prolapse	2/10	5/10	3/10
Performing SC/SC in the same hospital	*–*	5/10	2/10
Referring to another hospital for SC	2/10	*–*	1/10
Recurrent VVP after previous surgical treatment for VVP	–	10/10	–

No./no. of total respondents

*VVP* vaginal vault prolapse, *VSF* vaginal sacrospinous fixation, *SC* sacrocolpopexy (either laparoscopic sacrocolpopexy or robotic sacrocolpopexy)

**Table 3:** Top five factors influencing the choice of treatment given by gynecologists

Factors	Total number of points	Number of times mentioned
Recurrent prolapse	39	8
Comorbidity/surgical history	29	7
Age	14	6
Patient’s preference/fear of mesh	13	5
Body mass index	12	4
Higher POP-Q stage	8	2
Time till recurrence	8	2
Chronic pelvic pain	4	2
Type of complaints (e.g., urgency, pain)	4	1
Sustainability	4	1
Sexual function	3	1
Combination with rectopexy	2	1
Concomitant cystocele	1	1
No need for referral to another hospital	1	1

As this was an open-ended question to all participants, there are more than five factors included in this table

*POP-Q* pelvic organ prolapse quantification

### Patient-related factors

Patients’ medical /surgical history is the second most important factor for gynecologists to consider. All participants have stated that certain comorbidities or abdominal surgical history could be a reason for choosing VSF.

Quote 1:

“We perform a treatment to improve quality of life, which should never result in more morbidity thereafter. Sometimes it is inevitable of course, but you should do everything to minimize that risk. For some patients a vaginal procedure is much safer, e.g., patients with cardiac or pulmonary comorbidity.”

High age and high body mass index are factors that were interpreted differently by some participants. In some cases gynecologists prefer to perform a VSF in patients who are older (6 out of 10), e.g., over 80 or 85 years of age, as opposed to one gynecologist who prefers the SCP in that case. The same goes for higher BMI: 7 gynecologists opt for a VSF, compared with 2 gynecologists who chose a sacrocolpopexy.

Quote 2:

“A higher BMI is just the reason to do a sacrocolpopexy. It is a more effective treatment, because there is a lower chance of recurrence.”

Quote 3:

“When a patient has morbid obesity, and too much visceral fat, sacrocolpopexy can be a technically difficult procedure. In that case I would prefer a sacrospinous fixation.”

When considering surgical treatment for POP, regardless of the type of surgery, all gynecologists emphasize that patients should have enough bothersome complaints. The extent of bulge symptoms is not relevant in their choice between a VSF or SCP. However, the kind of complaints can be of importance when choosing a specific procedure for some participants. Urgency, severe constipation, or (chronic) pelvic pain are reasons to opt for a VSF instead of SCP, stated 4 respondents. One participant was hesitant to perform a VSF on a patient with pelvic pain or trigger points in the course of the pudendal nerve or the sacrospinous ligament.

Quote 4:

“When a patient has severe urgency complaints, I do not believe you should do a treatment with mesh. When someone has constipation, there is a chance of worsening after a sacrohysteropexy.”

Quote 5:

“When I palpate the sacrospinous ligament and it hurts, I counsel the patient differently. In the case of a primary vault prolapse, the patient has to have pelvic floor physical therapy first to relieve these pain symptoms, before I would perform a vaginal sacrospinous fixation.”

A lower or higher pelvic organ prolapse quantification (POP-Q) stage only matters for 2 participants. Three gynecologists pay attention to the vaginal length, they prefer sacrocolpopexy when the vaginal length is short in order to prevent a bridging suture or overcorrection of the prolapse in VSF.

### Surgery-related factors

The VSF can be performed with spinal analgesia, whereas general anesthesia is needed for a SCP. Three participants, who only perform VSF, said that it is an advantage to be able to perform a VSF with spinal analgesia. Five gynecologists do not believe that it is of importance or they leave it up to the anesthesiologist to decide whether general anesthesia can be administered; those are all gynecologists who perform the LSC in their own hospital.

Other surgery-related factors were subcategorized in to perioperative factors and postoperative factors. Nine participants stated SCP to be a more invasive procedure owing to a longer surgical time, a higher risk of complications, and the prolonged Trendelenburg position.

Postoperative factors that were mentioned are a lower recurrence rate or higher efficacy for the SCP (7 out of 10) and a higher chance of recurrent cystocele for the VSF (2 out of 10). One gynecologist said that de novo dyspareunia was a result of SCP, whereas 3 gynecologists said that it was a result of VSF. Two participants argued that de novo dyspareunia can be a result of both procedures.

Quote 6:

“Dyspareunia is always a bit tricky. We quite often see dyspareunia after sacrospinous fixation. On the other hand, in the case of pre-existent dyspareunia, you rather would not place a mesh.”

### Physician-related factors

Gynecologists who need to refer patients for a sacrocolpopexy all (4 out of 10) stated that they experience no barriers to doing so. One respondent did say that they preferred to treat a patient in their own hospital. Two participants said that most patients want to stay in their own hospital for surgical treatment for their prolapse, so they tend to choose the surgery that can be performed by their own gynecologist.

Quote 7:

“I do not experience a barrier in referring patients, as we have great collaboration in our region. However, patients prefer to be treated in their own hospital. So it seems they are the ones experiencing a barrier.”

Quote 8:

“The Dutch Medical Treatment Contracts Act (WBGO) demands that patients are informed of all treatment options, whether you can perform them yourself or not.”

### Uterine descent

All participants preferred a vaginal, uterine-preserving, treatment as first-choice surgery for primary uterine prolapse. The main reason is that they do not see an indication for the use of mesh for a first prolapse, as there are successful autologous tissue options. Comparable reasons were mentioned as the factors that play a role in the VVP, such as SCP is regarded as a more invasive procedure, carrying greater risks of mesh-related complications, and the risk of chronic pain. Which specific surgery they prefer is mainly based on their own clinical experience. Vaginal hysterectomy was only performed when there were other complaints, such as pre-malignancies, heavy menstrual bleeding, or the patient’s explicit wish to have their uterus removed.

A recurrent uterine prolapse is the main reason for considering a SHP. Two participants would prefer a SHP in the case of a recurrence. Six participants do not have a strong preference and would counsel a patient for a SHP or another vaginal treatment, which could be a VH, modified Manchester (MM), SSHP left-sided or bilateral, or uterosacral ligament suspension (USLS). Two gynecologists, who only perform vaginal surgery, would perform another vaginal treatment, either a SSHP after an MM or an MM after a SSHP. Similar factors to the post-hysterectomy vault prolapse are important to the participants, such as the patient’s health status. Anatomical characteristics of the uterine descent were of more interest than in the choice of the surgical treatment for VVP; e.g. the quality of the uterosacral ligaments or whether there is an elongated cervix. Furthermore, time until recurrence is important or whether the stitches through the SSL of the former VSF were torn out.

## Discussion

### Main findings

A qualitative study was performed to reveal the factors that are important for Dutch gynecologists in counseling their patients with apical prolapse. The results show that, for the treatment of primary VVP (i.e., no recurrence), some have a preference for VSF, some for SCP, and others have no preference. Recurrent VVP is the most important factor for choosing a SCP, for most gynecologists (8 out of 10). Second, comorbidity and surgical history are considered of relevance to both surgical procedures (7 out of 10). Further patient-related factors that play a role are age, BMI, and patients’ own preferences.

In the treatment for primary uterine prolapse all participants prefer a vaginal uterine-preserving treatment. In the case of a recurrent uterine prolapse, the surgical treatment preferences diverge greatly. The main reason for not choosing SCP is reluctance with regard to the use of mesh, as there are effective options with native tissue. Apparently most gynecologists conform to the Dutch guideline for the use of mesh implants in the treatment of POP and urinary incontinence, which states that abdominally placed mesh is more difficult and risky than vaginal native tissue procedures [16]. In the case of recurrent uterine descent or additional complaints (e.g., severe cervical dysplasia or heavy menstrual bleeding) a VH is preferred by the gynecologists in our study.

Age is a known risk factor for the development of POP, but age as a risk factor for recurrent POP is contradictory in the literature [17, 18]. A recent updated systematic review, however, defined younger age as a statistically significant risk factor for recurrent prolapse [19]. This last finding would suggest advising the surgical intervention with the lowest recurrence rates for younger patients. Some reviews and cohort studies suggest better outcomes for sacrocolpopexy [20–22]. However, this is not yet confirmed with long-term follow-up from RCTs directly comparing LSC with VSF [11].

Overweight and obese women are more likely to develop POP, compared with women with a BMI within the normal range [23]. For POP recurrence, BMI was not statistically significant as a risk factor. However, the authors state that a slight trend could be observed in the categorical variables BMI >30 versus BMI ≤30 [19]. Also a higher POP-Q stage before surgery (stage 3 or 4) was listed as a risk factor for POP recurrence [18, 19]. This was only mentioned by one participant to play a role in the selection of surgical treatment.

Some participants consider spinal analgesia to be beneficial compared with general anesthesia, especially for elderly patients. However, there seems to be no literature supporting this theory [24–26].

### Strengths and limitations

The main strength of this qualitative study is that the semi-structured interviews enabled us to examine this topic in more depth. We used the list of topics from the pilot interview and open-ended questions to explore factors and answers more in detail. This study only included Dutch gynecologists, so it mainly reflects Dutch clinical practice. However, all described surgical techniques are used internationally and therefore this study is still of interest to a broader audience.

During the interviews it became clear that participants had a different interpretation of the term “recurrence,” when talking about post-hysterectomy vault prolapse. Some gynecologists considered a vault prolapse after hysterectomy for POP to be a recurrence, whereas others thought a vault prolapse after previous surgical treatment for VVP to be a recurrence. This could have influenced the answers given by the gynecologists, although this misunderstanding was discovered during the first interview and the interpretation of “recurrence” was clarified at the beginning of each of the following interviews.

### Interpretation

Most gynecologists first set an indication for the type of surgery based on whether or not there is a recurrent prolapse. To come to a final decision, most gynecologists look at multiple factors combined. For example, this could lead to a 85-year-old patient with a recurrent vault prolapse, obesity, and multiple comorbidities undergoing a VSF instead of a sacrocolpopexy, whereas a 60-year-old patient with a recurrent VVP who has a very active lifestyle could undergo a sacrocolpopexy. When a sacrocolpopexy is the preferred option, it seems that the most important factors for the final choice between the procedures are (relative) contra-indications for sacrocolpopexy.

Gynecologists who do not perform the sacrocolpopexy themselves or in their own hospital find more factors important in favor of the VSF. They all see a benefit in the possibility of spinal analgesia, whereas in the other group only one participant stated this. It seems that they are more likely to perform a VSF in the case of an apical recurrence. RCTs investigating the optimal surgical treatments for apical prolapse have not demonstrated superiority of a certain surgery. Therefore, it seems reasonable that gynecologists consider certain patient-related and surgery-related factors to make a choice between treatments. Nevertheless, patients should be able to make a fully informed decision and doctors should aim to reduce practice pattern variability.

This study focuses on gynecologists’ preferences. Future research on patients’ preferences on the different kinds of treatments for VVP would be even more interesting. Furthermore, it would be helpful to incorporate factors that are truly of importance in a personalized decision aid to make the choice between procedures.

## Conclusions

Recurrent apical prolapse is the most important factor in advising patients on which treatment they should undergo for VVP or uterine descent. Also, patients’ comorbidities, surgical history, age, BMI, and the patient’s own preference are important factors. Gynecologists who do not perform the sacrocolpopexy in their own clinic are more likely to perform a VSF and find more reasons not to advise a patient to undergo a sacrocolpopexy. All participants prefer a vaginal surgery for a primary uterine prolapse.

### Supplementary information

Below is the link to the electronic supplementary material.

Supplementary file1 (PNG 59 KB)

## Data Availability

The data that support the findings of this study are available from the corresponding author upon reasonable request.

## Abbreviations

LSC: Laparoscopic sacrocolpopexy
LSH: Laparoscopic sacrohysteropexy
MM: Modified Manchester
POP: Pelvic organ prolapse
RSC: Robotic sacrocolpopexy
RSH: Robotic sacrohysteropexy
SCP: Sacrocolpopexy
SHP: Sacrohysteropexy
SSHP: Sacrospinous sacrohysteropexy
VH: Vaginal hysterectomy
VSF: Vaginal sacrospinous fixation (post-hysterectomy)
VVP: Vaginal vault prolapse
