# Supplementary figures and images for: Gynecologists’ perspectives on surgical treatment for apical prolapse: a qualitative study
Source: Int Urogynecol J. 2023 Jul 1;34(11):2705–12. doi: 10.1007/s00192-023-05587-1 (PMC10682281; doi:10.1007/s00192-023-05587-1)

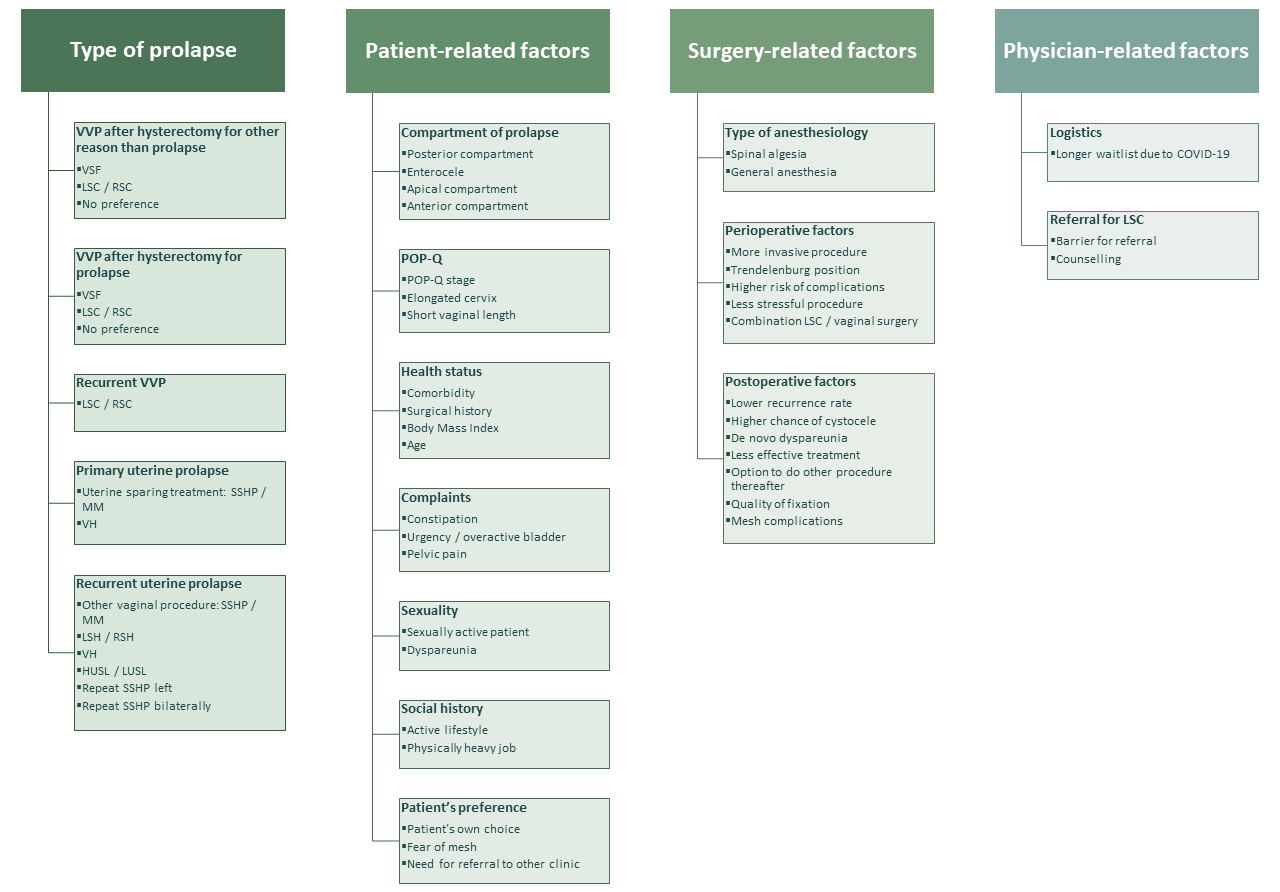

Supplement: Supplementary file 1 — Supplementary file1 (PNG 59 KB) [file 192_2023_5587_MOESM1_ESM.png]
